# New Insights Into the Interplay Among Autophagy, the NLRP3 Inflammasome and Inflammation in Adipose Tissue

**DOI:** 10.3389/fendo.2022.739882

**Published:** 2022-03-31

**Authors:** Liyuan Zhu, Ling Liu

**Affiliations:** ^1^ Department of Cardiovascular Medicine, The Second Xiangya Hospital, Central South University, Changsha, China; ^2^ Research Institute of Blood Lipid and Atherosclerosis, Central South University, Changsha, China; ^3^ Modern Cardiovascular Disease Clinical Technology Research Center of Hunan Province, Changsha, China; ^4^ Cardiovascular Disease Research Center of Hunan Province, Changsha, China

**Keywords:** inflammation, autophagy, inflammasome, NLRP3, adipose tissue

## Abstract

Obesity is a feature of metabolic syndrome with chronic inflammation in obese subjects, characterized by adipose tissue (AT) expansion, proinflammatory factor overexpression, and macrophage infiltration. Autophagy modulates inflammation in the enlargement of AT as an essential step for maintaining the balance in energy metabolism and waste elimination. Signaling originating from dysfunctional AT, such as AT containing hypertrophic adipocytes and surrounding macrophages, activates NOD-like receptor family 3 (NLRP3) inflammasome. There are interactions about altered autophagy and NLRP3 inflammasome activation during the progress in obesity. We summarize the current studies and potential mechanisms associated with autophagy and NLRP3 inflammasome in AT inflammation and aim to provide further evidence for research on obesity and obesity-related complications.

## 1 Introduction

Owing to the rapid socioeconomic development and increasingly obesogenic environments, obesity has become prevalent worldwide in recent decades (1, 2). Obesity is a serious health issue. According to the latest national surveys based on Chinese criteria of obesity, the number of obese (body mass index (BMI) ≥ 28 kg/m^2^) adults has more than quadrupled and the number of overweight (BMI 24-27.9 kg/m^2^) has more than doubled in China (3–5). Data also showed that 16.4% of Chinese adults were obese and another 34.3% were overweight (6).

Obesity increases the risk of several inflammation-related complications, including type 2 diabetes and insulin resistance, cardiovascular diseases, hepatic steatosis and several types of cancer. Obesity and its complications involve plenty of etiological mechanisms, including disproportionate or unbalanced food intake and energy expenditure, and a complex interplay between genetic and environmental factors that have effects on hemodynamic, neurohormonal, and metabolic regulation, leading to oxidative stress, inflammation, apoptosis and lipotoxicity (7). The pathogenic progression of obesity-related complications is exacerbated by persistent adipose tissue (AT) inflammation (8, 9). Current approaches to obesity management are mainly containing lifestyle interventions (physical exercise and dietary modification) and non-lifestyle interventions (weight-loss medications or surgery). One recent review summarizes that natural and pharmaceutical agents display promise in alleviating endoplasmic reticulum stress and maladaptive unfolded protein response which improves obesity pathogenesis and management (10). How to effectively reduce AT inflammation is a challenging task in obesity management.

Recent research support altered (enhanced or inhibited) autophagy is major process regulating cellular metabolism and energy homeostasis, which has integrated into AT inflammation (11, 12). Obese individuals exhibited elevated release of inflammatory cytokines and raised infiltration of macrophages in WAT (white adipose tissue), including visceral white adipose tissue (vWAT) and subcutaneous white adipose tissue (sWAT) (13). The NOD-like receptor protein 3 (NLRP3) inflammasome has recently been demonstrated to detect nonmicrobial danger signals in dysfunctional AT and involved in obesity-related inflammation (14, 15). Moreover, WAT resists obesity-related inflammation through compensatory enhancement of autophagy. Thus, WAT from obese individuals presented increased levels of autophagic markers, autophagy-related genes (ATGs), microtubule-associated protein 1A/1B-light chain 3 (LC3), p62, and Beclin-1, at either gene and/or protein levels (11, 16, 17). Here, we focus on the mutual regulation and cellular mechanism between NLRP3 inflammasome and autophagy in AT during obesity.

## 2 Adipose Tissue Composition and Inflammation

To store extra energy and prevent ectopic lipid accumulation, AT mass expanses in two ways: hypertrophy (increasing the size of existing adipocytes) or hyperplasia (the differentiation of preadipocytes into adipocytes) (18). The quantity of adipocytes in AT is determined early in infancy and remains constant throughout maturity. The balance between hypertrophy and hyperplasia in adipocytes has a significant impact on energy metabolism in AT (19). AT is composed of various cell types in adult mammals, including adipocytes, macrophages, vascular endothelial cells, fibroblasts, preadipocytes, and other immune cells (20).

Preadipocytes develop into new adipocytes in response to sustained caloric excess, contributing to AT enlargement. For example, adipocytes with smaller lipid droplets can store lipids, convert excess free fatty acids (FFA) to triglycerides, and maintain an insulin-sensitive state. AT expansion by increasing adipogenesis can decrease cellular inflammatory cytokines and the number of hypertrophic adipocytes (21). Newly differentiated adipocytes have the potential of hypertrophy, which can grow to hundreds of microns in diameter (18). Larger adipocytes expand to the oxygen diffusion limit, resulting in cellular hypoxia. The small adipocytes gradually increase in size and contact surrounding cells, resulting in the increased extracellular matrix and mechanical pressure. When hypoxia is combined with mechanical and metabolic stress, small adipocytes enhance adipogenesis, lowering hypoxic stress and consequent inflammation in AT. It revealed that adipogenic capacity from preadipocytes enabled WAT in obese individuals to perform normal physiological functions (22).

As an endocrine organ, AT produces various proinflammatory cytokines and integrates immune signaling in dysfunctional metabolic status (23). Some cytokines, including tumor necrosis factor (TNF)-α, interleukin (IL)-1β, IL-6, IL-8, and monocyte chemotactic protein-1 (MCP-1), also indicated metabolic dysfunction of AT (24). Individuals with more hypertrophic adipocytes typically had higher levels of proinflammatory cytokines and lower levels of adiponectin and IL-10 (25, 26). Hypertrophic adipocytes drove collagen deposition and fibrosis, resulting in AT remodeling and insulin resistance (27–30). It has been suggested that FFA promotes inflammation mediated by Toll-like receptors (TLRs) in adipocytes and macrophages. FFA activated TLR 4/TLR 2, stimulated nuclear factor kappa B (NF-κB) and Jun amino-terminal kinase (JNK) signaling, induced the expression of inflammatory cytokine genes, such as TNF-α and IL-6, and aggravated insulin resistance in adipocytes and macrophages (31, 32). P38 mitogen-activated protein kinase (MARK) and Jnk were upregulated and activated in vWAT with increased numbers of hypertrophic adipocytes. The extent to which p38 MARK and Jnk were phosphorylated, particularly in vWAT, corresponded with fasting levels of triglycerides, insulin, and glucose (33). Another study confirmed that mice with AT-specific *Jnk1* deletion were protected against diet-induced insulin resistance and inflammation (34). These results indicated that TLRs were connected to cytokine activation *via* NF-κB and JNK in adipocytes and macrophages.

**Figure 1:**
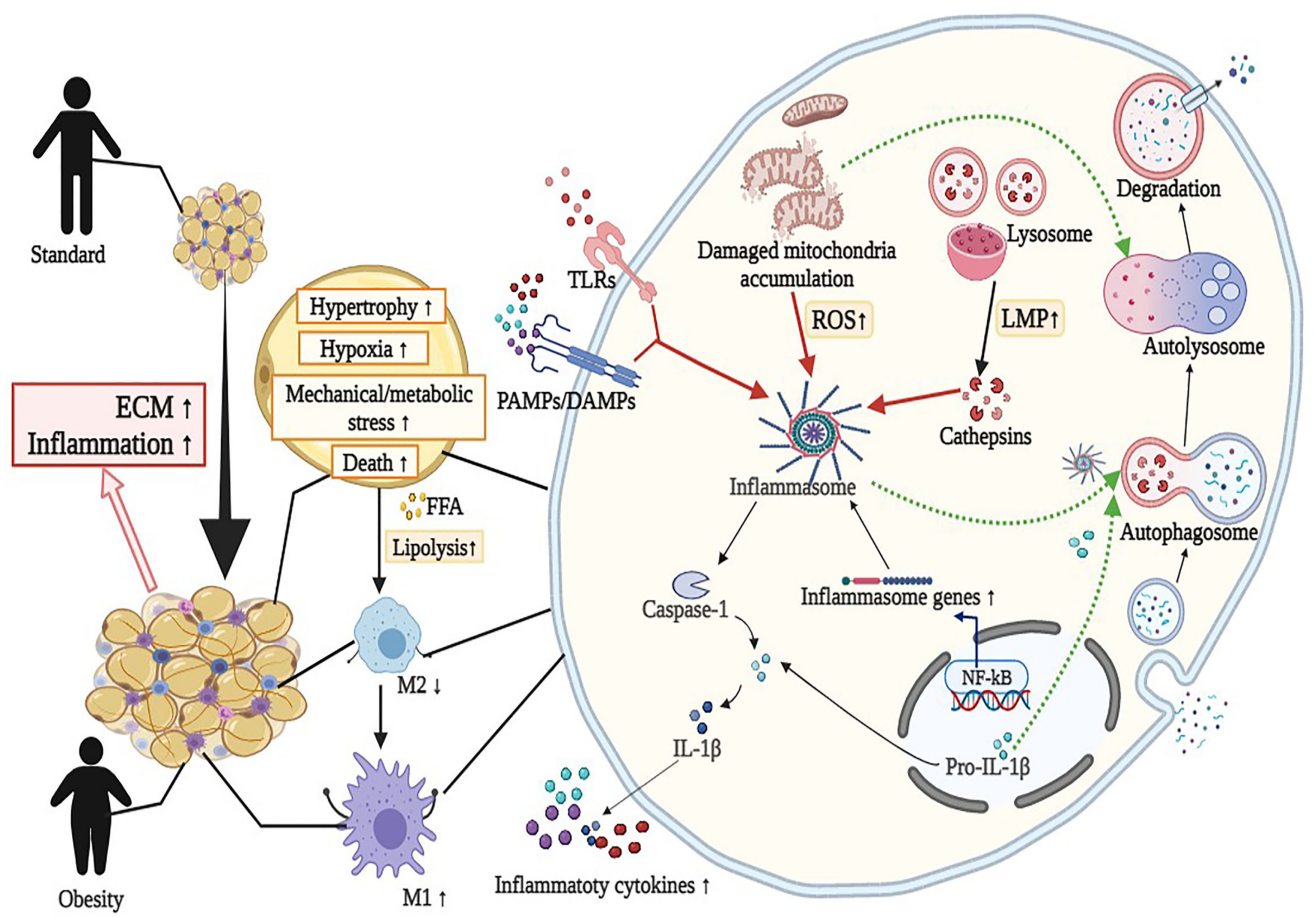
Overview of the interaction between autophagy and NLRP3 inflammasome in adipose tissue inflammation (mainly adipocytes and macrophages). Activation of NLRP3 inflammasome by diverse metabolic stimuli (such as LPS, cathepsins, and mitochondrial dysfunction) *via* TLRs or PAMPs/DAMPs leads to metabolic and immune dysregulation, including insulin resistance, macrophage polarization (M2 to M1), hypoxia, impaired adipogenesis and increased fibrosis in adipose tissue. Detailed descriptions and explanations for each alteration can be found in Section 2 and Section 3. DAMPs, damage-associated molecular patterns; ECM, extracellular matrix; FFA, free fatty acids; IL-1β, interleukin 1β; LMP, lysosomal membrane permeabilization; LPS, lipopolysaccharide; ROS, reactive oxidative species; PAMPs, pathogen-associated molecular patterns; TLRs, Toll-like receptors. Image created with BioRender.com.

Crosstalk between adipocytes and adipose tissue macrophages (ATMs) leads to chronic inflammation and accelerates the inflammatory process in AT (35). First, increased FFA and chemotactic factors, for example MCP-1, derived from adipocytes can enhance the accumulation and transition of proinflammatory M1 macrophage subsets rather than anti-inflammatory M2 macrophage subsets in the expanded AT (36). For example, AT from obese mice with high-fat diet (HFD) represented increased F4/80 and *CD11b+* macrophages and elevated IL-6 and MCP-1 levels. The high double-positive *CD11b+* and F4/80 macrophages and inflammatory cytokines were reduced in mice through docosahexaenoic acid supplement (37). Another study showed that n-3 PUFAs added to mice with HFD prevented macrophage chemotaxis, stimulated M2 polarization, and suppressed M1 polarization in AT from *in vitro* and vivo experiments (38). Second, with increased volume and number of adipocytes, hypertrophic adipocytes have to face heavier mechanical stress and hypoxia, which further exacerbated ATMs-related inflammation (39, 40). Selective *Jnk* insufficiency in macrophages decreased ATM infiltration and maintained insulin sensitivity in mice fed HFD. Hypertrophic adipocytes triggered the release of proinflammatory cytokines and then attracted more macrophages to AT through increasing the productions of reactive oxidative species (ROS) and endogenous redox stress (41–44). Notably, ATMs delivered proinflammatory signals to other organs, indicating a link between obesity and secondary organ damage, such as that in the liver, skeletal muscle, and pancreas (45).*In vivo* imaging studies showed that ATM migrated from the periphery of apoptotic adipocytes, such as crown-like structures, and resided in the cellular interstitium of other tissues (46) (**Figure 1**).

## 3 Obesity and Autophagy

Autophagy is an evolutionarily conserved process in all eukaryotes that transports damaged proteins and organelles to the lysosome for degradation. Autophagy is intimately involved in the development of obesity and obesity-related metabolism (47, 48) (**Figure 1**). Excessive autophagy is hypothesized to be another form of cell death known as autophagy-related cell death (49).

### 3.1 Autophagy and WAT Inflammation

Autophagy is associated with innate and adaptive immunity, the elimination of pathogens and intracellular waste, and the regulation of immune functions in immune and nonimmune cells, such as antigen presentation and cytokine synthesis and secretion (50, 51) (**Figure 1**). The outcome of autophagy is determined by the type of stimulus, the microenvironment, cell types, and other biological factors (52). Consistent with its role in pathogen degradation, the activation of pattern recognition receptors such as TLRs or nucleotide oligomerization domain-like receptors (NLRs) initiated autophagy by endogenously recognizing and binding microbial components, such as pathogen-associated molecular patterns (PAMPs) or damage-associated molecular patterns (DAMPs) (53). Once activated, autophagy suppresses inflammation by blocking the generation and secretion of proinflammatory molecules, including IL-1β degradation *via* autophagosomes and the direct inhibition of inflammasomes (53–56) (**Figure 1**).

The variations in autophagy between vWAT and sWAT must be investigated independently in obesity (57, 58). Kovsan et al. discovered that patients with vWAT accumulation had significantly higher mRNA levels of ATG5, LC3-I, and LC3-II than those with peripheral obesity. The researchers concluded that autophagic genes and proteins were expressed more abundantly in vWAT than in sWAT, independent of BMI or glycemic status (17). Numerous studies have examined the link between autophagy in WAT and adipocyte size in obesity (16, 17, 59). Adipocyte size was strongly correlated with ATG expression (16, 17). However, not all research has demonstrated a clear correlation between adipocyte size and adipocyte autophagic flux (16, 17, 59). Different methodologies may account for these discrepancies. The link between autophagy flux and hypertrophy in adipocytes should be further examined.

Compared to normoglycemic and lean individuals, AT from obese ones was characterized by increased autophagy, proinflammatory cytokines, and macrophage infiltration (13). *In vitro*, hypertrophic 3T3L1 adipocytes had decreased autophagy and increased inflammation. 3-Methyladenine, an autophagy inhibitor and lysosomal blocker, increased inflammatory cytokine expression (12). The outcome validated the connection in the opposite direction. The specific mechanism by which WAT alleviates nutritional stress at the molecular and cellular levels has not been determined.

### 3.2 Autophagy Flux Detection of WAT Fraction

Evaluating alterations of autophagy requires analyzing the autophagy flux of different cell types in WAT. Several studies have examined autophagy in mature adipocytes and stromal vascular fractions (SVFs) (11, 17, 60). Expressions of ATGs have been shown in mature adipocytes. The difference between the SVF and adipocytes depended on the analyzed ATGs, although it was unclear which component of AT influenced the overall autophagy level. Kovsan et al. discovered that visceral adipocytes had much higher gene and protein expressions of ATG5 and LC3 than subcutaneous adipocytes (17). Jansen et al. demonstrated that ATG5 and ATG7 expression levels were reduced in the SVF and adipocytes of the vWAT and sWAT of healthy individuals. However, the researchers discovered that ATG1 and ATG16L expression levels were lower in mature adipocytes than in the SVF, indicating the opposite of previously observed (11). On the contrary, Rodriguez et al. showed no significant change in Beclin-1, ATG5, or ATG7 expression between visceral adipocytes and the SVF in obese patients. Although not statistically significant, ATG7 expression tended to be higher in the SVF than in adipocytes (60). Increased autophagy in WAT was related to the metabolic state. Clinical studies have shown that the gene and protein levels of autophagic markers in sWAT and vWAT are favorably linked with plasma triglycerides, cholesterol, FFA, and the insulin resistance index but negatively correlated with adiponectin (11, 17). It was difficult to identify special cell clusters of WAT engaged in autophagy when confronted with various stress or metabolic signaling in obesity. Further study of autophagic activity should focus on cells directly isolated from AT in different metabolic states.

The polarization of macrophages and function-related autophagy in macrophages are critical for WAT immunological homeostasis. WAT of *Atg7* mutant mice fed HFD had increased the infiltration and proportion of proinflammatory M1 macrophages and impaired systemic insulin sensitivity. Inhibition autophagy *in vitro* experiment was similarly characterized by increased ROS and IL-1β in macrophages (61–63). Recent research showed that *Lc3* levels of macrophages in WAT from genetically modified obese mice were much higher than that of diet-induced obesity (DIO) animals. Additionally, the data indicated that the microenvironment of WAT could influence local ATMs by increasing autophagic flux (64). However, given that autophagy is considered to inhibit the inflammatory response (39), increased inflammation in WAT in genetically modified obese mice is perplexing.

### 3.3 WAT Autophagy: Animal Models

Due to the convenience and availability of animal models, systemic metabolic changes related to autophagy in WAT come from mice studies (**Table 1**). Genetic deletion of *Atg7* or *Atg5*, which were essential autophagy genes in adipocytes, resulted in the impaired differentiation of white adipocytes. *Atg7* or *Atg5* knockout mice exhibited decreased diet-induced weight gain, increased WAT volume, and improved insulin sensitivity (65, 66). Additional studies indicated that *p62* affects energy metabolism in AT by regulating the activity of mitochondria. *P62* deficiency in adipocytes resulted in decreased mitochondrial activity. The activation of *p38* targets was reduced by silencing *p62*, impacting signaling molecules that governed mitochondrial activity (67). *Bif-1* is a protein that induces membrane curvature and is involved in controlling autophagy. Bax-interacting factor 1(*Bif-1*) deficiency increases the volume of AT and contributes to the development of insulin resistance. Obesity caused by *Bif-1* deficiency decreased the expression of proteins involved in the autophagy-lysosomal pathway in adipose tissue, including *Atg9a* and *Lamp1* (68). If adipogenesis was viewed as a mechanism for the healthy expansion of WAT, *Atg-*knockout mice with favorable metabolic profiles appear contradictory. These findings indicated that *Atg5* or *Atg7* deletion in mice enhanced oxidation during metabolic activity and reduced browning and fibrosis in WAT (65, 66, 71). The capacity of increased heat production and oxygen consumption may help to explain animals remaining in an insulin-sensitive state, even when WAT adipogenesis is decreased. The aforementioned findings indicated that autophagy was required for hyperplasia. Autophagy activation in AT may be necessary for adipogenesis and healthy expansion in obesity. Although data from autophagic gene knockout animal models have been acquired, the specific regulation of autophagic processes in obesity and related metabolic disorders is unclear.

**Table 1:** Animal studies about autophagy and inflammation in obese models.

Reference	Groupings	Diet types/Intervention	Samples	Results (compared to the control group)
Zhang Y et al. (65, 66)	*ATG7* ^–/–^ transgenic C57BL/6 mice	HFD (60 kcal% fat) for 8 w beginning at 8 w of age	Gonadal WAT	β-oxidation, basal physical activity, LC3-I, P62 ↑; Lipolysis, leptin, triglyceride, cholesterol ↓; Absence of LC3-II
Yoshizaki et al. (12)	GFP-LC3 transgenic C57BL/6 mice	NCD (13.5% fat) or HFD (60% fat) for 16 w beginning at 8 w of age	Epididymal AT	LAMP1, LAMP2, and ATG5 ↓; LC3-II, MCP-1, IL-6, and IL-1β ↑
Mueller et al. (67)	*P62^–/–^* transgenic C57BL/6 mice	NCD (5.6% fat) or HFD (58% kcal fat)	BAT	Impaired mitochondrial function; Activation of p38 targets ↓
Liu Y et al. (68)	Bif-1^−/−^ and wildtype transgenic C57BL/6 mice	NCD (18% kcal from fat) or HFD (55% kcal from fat;) at 6 w of age	WAT	P62, LC3-II/LC3 ↑; Bif-1, ATG9a, LAMP1 ↓
Mizunoe Y et al. (69)	*Ob/ob* and wild-type C57BL/6 mice	NCD (10% kcal from fat) or HFD (60% kcal from fat;) for 4 w beginning at 6 w of age	Epididymal AT	Obese AT: CTSB, LC3-I/LC3-II, P62 ↑;CTSL ↓; ATG5 and Beclin1 unchanged
Nunez CE et al. (70)	Male Swiss mice	NCD (10% kcal from fat) or HFD (60% kcal from fat;) for 8 w beginning at 4 w of age	Epididymal AT	Beclin1, P62, CHOP, phospho-JNK ↑; Phospho-mTOR ↓

AT, adipose tissue; WAT, white adipose tissue; BAT, brown adipose tissue; NCD, normal chow diet; HFD, high fat diet; T2D, type 2 diabetic; TNF, tumor necrosis factor; IL, interleukin; CTSB, cathepsin B; CTSL, cathepsin L; LAMP, lysosomal associated membrane protein.

Research needs to explore the WAT autophagy state to elucidate the mechanisms of WAT dysfunction in obesity. There was impaired autophagy in WAT in DIO mice (12, 69). It reported that *Lc3-II* and *p62* protein levels were increased in the vWAT of DIO mice, while *Atg5* or *Beclin-1* levels remained unchanged. Autophagic flux and autophagosome formation were enhanced in vWAT, and vWAT lysosomal function was impaired in DIO mice (69). Another study reported that DIO mice presented inhibited autophagy, but *Lc3-II* immunofluorescence analysis was not performed (12). In addition, Nuñez et al. described decreased levels of phosphorylated mTOR and increased *Beclin-1* and *p62* in DIO mice compared to lean mice (70), and there were reduced numbers of autophagosomes in AT of calorie-restricted obese mice compared with calorie-restricted lean mice. It was concluded that obesity and caloric overfeeding are associated with the defective regulation of autophagy in AT.

Another related issue was the exact step that regulated the autophagic process and was affected by DIO. Yoshizaki et al. showed that autophagy is impaired at an early stage of autophagosome formation in DIO mice and that lysosomal inhibition led to LC3-II accumulation (12). In contrast, Mizunoe et al. reported an increase in diet-induced autophagosome formation, an increase in *Lc3-II* and *p62* after lysosomal inhibition, and the dysregulation of protease lysosomes, suggesting that the autophagic process may be gradually attenuated in DIO animals (69). Similar to other studies, these differences might result from different experimental approaches and uniform criteria were required to determine the autophagic status (52). Another problem was differences in the timing of diets from study to study, and the effects on autophagy may differ (12, 69, 70). Notably, there were inconsistent interpretations of the autophagic state in different studies. For example, it has been suggested that elevated *p62* attenuates autophagy (69). It has also been suggested that elevated *p62* enhances autophagy (70). It is difficult to draw a definite conclusion in these cases.

### 3.4 WAT Autophagy: Human Clinical Study

The convenience of obtaining AT compared to that of liver or muscle from humans has ensured research in obese individuals (**Table 2**). Regarding human studies, the overall trend was that WAT autophagy was increased in obese individuals (11, 13, 16, 17, 70, 72). Autophagy markers (Beclin-1, ATG5, ATG12, ATG7, LC3-I, LC3-II, and p62) in obese patients had higher mRNA or protein levels than those in lean individuals, and mammalian target of rapamycin (mTOR) expression was decreased, along with sWAT or vWAT (11, 13, 16, 17, 70, 72). Notably, not all studies analyzed the same autophagy markers, and the expression of these autophagy markers was analyzed independently in each study. Given the inconsistent conclusions on autophagic status in the WAT of DIO mice and obese subjects, it is necessary to confirm whether autophagic flux is accompanied by increased expression of autophagic proteins related to substrate recognition and autophagosome formation, such as LC3 or p62. There are two opposing situations: increased autophagic flux and impaired lysosomal degradation, leading to autophagosome accumulation. Autophagic flux assays could distinguish between these two conditions (52). Thus, *in vitro* experiments under lysosomal inhibitor conditions revealed enhanced autophagic flux in sWAT and vWAT from obese patients and DIO mice, and the findings suggested consistent WAT autophagic gene or protein expression in obesity (17, 69).

**Table 2:** Clinical studies about autophagy and inflammation in obese individuals.

Reference	Groupings	Diet types/Intervention	Samples	Results (compared to the control group)
Nunez CE et al. (70)	9 obese-nondiabetic and 6 obese-diabetic subjects	Bariatric surgery	Subcutaneous AT	Body mass, Beclin1, autophagosomes, TNF-1a, IL6, IL-1β ↓
Camargo A et al. (72)	39 obese subjects with metabolic syndrome	Four dieting models: HSFAD, HMUFAD, LFHCCD with longchain n-3 polyunsaturated fatty acids (n-3) or placebo (LFHCCD) for 12w	Subcutaneous AT	HMUFA diet: Beclin1 and ATG7 ↑; LFHCC and LFHCC n-3 diet: Caspase-3, Caspase7, HOMA-IR ↑
Soussi H et al. (16)	Middle-aged obese or overweight subjects	Bariatric surgery	Subcutaneous AT	Obese adipocytes: P62 ↑; barely detected LC3-II with lysosome inhibitor; Nonobese adipocytes: LC3-II accumulation with lysosome inhibitors ↑
Jansen HJ et al. (11)	Healthy lean and obese subjects at age of 30-70 years old	–	Visceral and subcutaneous AT	LC3-II ↑; positively correlated with systemic insulin resistance and morphological characteristics of AT inflammation; Obesity with 3-methylalanine: proinflammatory gene expression, IL-1β, IL-6, IL-8 ↑
Kosacka J et al. (13)	60 lean and obese subjects with (n = 20) or without T2D (n = 20)	Open abdominal surgery (cholecystectomy, abdominal hernia, gastric sleeve, Roux-enY gastric bypass surgery or explorative laparotomy)	Visceral and subcutaneous AT	Obesity with T2D: autophagy, apoptosis, LC3 ratio, TNF-α, IL-1β, IL-6, IL-10 ↑; IL-10 ↓; Lean and nondiabetic: LC3 cannot detect in AT
Kovsan, J et al. (17)	Obese and nonobese subjects	Elective abdominal surgery (bariatric surgery or cholecystectomy)	Omental and subcutaneous AT	ATG5, LC3-I and LC3-II were higher in Omental AT than subcutaneous AT among obese subjects, with intraabdominal fat accumulation; Obesity with lysosome inhibitors: autophagy genes, autophagosomes, autophagic flux ↑

AT, adipose tissue; WAT, white adipose tissue; T2D, type 2 diabetic; TNF, tumor necrosis factor; IL, interleukin; HSFAD, high-saturated fatty acid diet; HMUFAD, high-monounsaturated fatty acid diet; LFHCCD, low-fat, high-complex carbohydrate diets; HOMA-IR, homeostatic model assessment for insulin resistance; CTSB, cathepsin B; CTSL, cathepsin L; LAMP, lysosomal associated membrane protein.

Studies on WAT autophagy have also been performed in humans with weight-loss operations, confirming that autophagy levels were elevated in obese states (70, 72). Nuñez et al. showed decreased Beclin-1 and autophagosomes in the sWAT of obese patients after bariatric surgery with or without diabetes (70). Some investigators have suggested that metabolic improvements after bariatric surgery are associated with sWAT autophagy. Soussi et al. indicated that autophagic flux in adipocytes was impaired in obese patients, while autophagic flux was restored after bariatric surgery (16). The study only compared the levels of LC3-II before and after surgery in response to lysosomal inhibition. Autophagic flux was not detected in the presence of lysosomal inhibition. In this context, autophagy plays a protective role against nutrient metabolism-related stress. However, the specific expression of ATGs in response to metabolic changes caused by surgery has not been investigated, and the function of operation-induced vWAT autophagy in systemic responses is unclear.

## 4 Obesity and NLRP3 Inflammasome

Unhealthy expansion of AT referred to the accumulation of inflammatory factors and destruction of cellular homeostasis. The canonical NLRP3 inflammasome consists of the NLRP3 receptor, the adaptor molecule apoptosis-associated speck-like protein with a CARD (ASC), and Caspase-1. NLRP3 inflammasome interacted with the adaptor protein ASC and then recruited inflammatory Caspase-1 to the complex, subsequently oligomerized into pentameric inflammasomes. Caspase-1 was a common effector molecule that cleaved the inactive precursors of IL-1β and IL-18 into their mature forms, and these factors are then secreted from cells. Transcription factor NF-κB was the most crucial signal in the activation of NLRP3 inflammasome. One study investigated mice fed HFD and found increased activity of *NF-κB* and systemic inflammation. Inflammasome-induced pyroptosis was alleviated by blocking *NF-κB/Gsdmd* signaling in AT of mice (73). In contrast to other inflammasomes, NLRP3 inflammasome may be activated in response to various stimuli (74). Evidence showed that ROS, lysosomal membrane permeabilization (LMP), and mitochondrial dysfunction were involved in NLRP3 inflammasome activation (75–77) (**Figure 1**).

A recent systematic review revealed increased NLRP3 and IL-1β in the sWAT and vWAT of obese individuals or mouse models (78). For example, increased gene expression of NLRP3 and its products IL-1β and IL-18 was found in the vWAT of metabolically unhealthy obese individuals compared to lean or metabolically healthy obese individuals or healthy controls (79). Alternatively, obese individuals with increased visceral fat ratios or increased vWAT and sWAT had elevated IL-1β, Caspase-1, and NLRP3 gene expression (80). A study in mice showed that *Nlrp3* inflammasome recognized lipotoxicity-associated elevations in intracellular ceramide to trigger Caspase-1 cleavage in macrophages and AT, and the expression of inflammasome components in sWAT was positively linked with ceramide levels. In obese mice, knocking down *Nlrp3* decreased *Il-18* expression and effector T cells and increased naïve T cells in AT (81). Adipocytes but not SVF showed an increase in NLRP3 and IL-1β expression in the sWAT of obese females (82). Obese and type 2 diabetic patients exhibited decreased gene expressions of IL-1β and NLRP3 in sWAT and increased insulin sensitivity after diet intervention or exercise (81). Likewise, weight loss by bariatric surgery also reduced gene expression and secretion of IL-1β in the AT of humans and animals (81, 83–85). Other studies showed that inflammasome activators were decreased, and inflammasome inhibitors were increased after bariatric surgery (86–88). It was unclear whether these changes directly caused NLRP3 inflammasome declines in AT.

### 4.1 NLRP3 Inflammasome Regulation in WAT

The senescent WAT was associated with an inappropriate expansion of adipocytes, insulin resistance, and dyslipidemia (89). Inflammasomes in senescent WAT, each of which had its priming and stimulus, such as gut-derived endotoxin, adipocytokines, lipid metabolites, and mitochondrial dysfunction, played an essential role in chronic inflammation and insulin resistance (90–95). Adipocytes differentiate, hypertrophy, and die in response to nutritional status or environmental variables. Caspase-1 and IL-1β were expressed dynamically during the differentiation of adipocytes (96). Stienstra et al. found that Caspase-1 inhibition increased genes expression of adipogenesis in adipocytes, including adiponectin and peroxisome proliferator-activated receptor γ, and inhibited IL-1β but not IL-18 production *in vitro*. *Caspase-1-* o*r Nlrp3-* promoted adipogenesis and improved AT function and insulin sensitivity in mice (96). A recent study showed that NLRP3 inflammasome was activated by LPS and palmitic acid, which contributed to adipogenesis and, conversely, inhibited the osteogenesis of stem cells *in vitro* (97). The reason for these differences may be the different activators of NLRP3 inflammasome or cell types. NLRP3 inflammasome was implicated in the downregulation of adipogenesis in the sWAT of obese adolescents (80). Impaired adipogenesis may affect *de novo* adipocyte recruitment and lead to preexisting hypertrophy of adipocytes. Studies showed that *Nlrp3-* or *Caspase-1* prevented hypertrophy of adipocytes in DIO mice (81, 98). However, the mechanism of energy consumption requires a detailed examination of inflammasomes in the process of adipogenesis and lipolysis.

Autophagy is a conserved, lysosome-mediated catabolic mechanism that is responsible for degradation and recycling. Cysteine cathepsins in the lysosome are a cluster of compensatory proteases involved in various cellular processes such as proteolytic degradation through crossing or overlapping signaling pathways. Lysosomal cysteine cathepsins include cathepsins B (CTSB), C, F, H, K, L, O, S, V, and Z (99). Some researchers showed that the mRNA and serum levels of CTSS were positively correlated with BMI and were decreased by weight loss (100). Others demonstrated that CTSK, CTSB, and CTSL were expressed in AT and were elevated in obese humans and mice models (69, 101–103). CTSB has been implicated as a modulator of NLRP3 inflammasome activation through the release of the lysosomal enzyme due to LMP (77). According to one study, the overexpression of *Ctsb* in obese mice increased Caspase-1 *in vivo* and *in vitro* (69). Mizunoe et al. discovered that *Ctsb* overexpression inhibited the expression of Perilipin-1 in 3T3L1 adipocytes. *Ctsb* overexpression resulted in lipolysis and metabolic dysfunction in 3T3L1 adipocytes (104). Enhanced autophagy in *db/db* mice fed HFD showed the accumulation of autophagosomes and an increased ratio of *Lc3-II* to *Lc3-I* with low-grade systemic inflammation (105). Reduced protein expression of Perilipin-1 was related to the activation of inflammatory responses in obese individuals (106). Understanding the specific inflammasome pathways activated by cysteine cathepsins will be important for obesity-related metabolic diseases.

### 4.2 NLRP3 Inflammasome and AT Remodeling

Chronic and systemic inflammation underlined immune activation during obesity. Dysfunctional AT exhibits impaired angiogenesis, altered extracellular matrix (ECM) remodeling and fibrosis (43). One study showed that blocking the expression of NLRP3 inflammasome reduced AT inflammation and significantly attenuated fibrosis by decreasing the production of IL-1β (107). In addition, researchers have revealed that NLRP3 inflammasome in visceral adipocytes is regulated by exogenous (LPS, aluminum or TNF-α) and endogenous (ATP or TNF-α) factors and hypoxia. NLRP3 gene silencing reduced the expression and release of inflammatory markers, such as IL-1β and IL-6, IL-8, and TNF-α (107). NLRP3 inhibition in AT attenuated the expression of essential molecules involved in ECM deposition and fibrosis, including different collagens and proteases, such as collagen type I alpha 1 chain 1 (COL1A1), COL4A3, COL6A3, MMP2, and MMP9 (107, 107).

In addition, activation of NLRP3 inflammasome in AT exacerbated fibrosis, restricted the healthy expansion of adipocytes and increased circulating levels of FFA during obesity (28, 107). Such as palmitate decreased active AMP-activated protein kinase, blocked the unc-51–like kinase-1 autophagy signaling cascade, and reduced glucose tolerance and insulin sensitivity in HFD mouse models (108). Genes knockdown of *Nlrp3* and *Tlr4* prevented diet-induced AT fibrosis in mice by modulating upstream factors of the inflammatory response in immune cells (109). There had been few studies on inflammation in brown AT, but there was evidence that inflammation could impair thermogenesis and exacerbate the whitening of BAT (40). Mice with *Atgl* knockout exhibited increased whitening of BAT and the induction of *Nlrp3* inflammasome expression (109). These results provided a helpful framework to understand the pathogenesis of obesity-associated diseases. A better understanding of the involvement of NLRP3 inflammasome pathway will inspire the development of therapeutics for reducing collagen deposition and fibrosis in AT.

### 4.3 NLRP3 Inflammasome and Obesity-Associated Liver Disease

Inflammasome activation also has been recently recognized to play a critical role in the development of obesity-associated liver disease. The histological evidence of hepatic steatosis from 9-month-old DIO *Nlrp3-* mice suggests that compared to WT mice, the ablation of NLRP3 resulted in reduction in hepatic steatosis. Obesity-related inflammasome activation in AT and liver was prevented, and insulin signaling was improved in *Nlrp3-* mice (81). Another study generated global and myeloid cell-specific conditional mutant *Nlrp3* knockin mice, resulting in a hyperactive *Nlrp3*. It demonstrated that global and myeloid-specific NLRP3 inflammasome activation resulted in severe liver inflammation and fibrosis while identifying a novel mechanism of NLRP3-mediated liver damage (110).

## 5 Interaction Between Autophagy and NLRP3 Inflammasome

There is a reciprocal regulatory relationship between autophagy and inflammasome activation. Not only does autophagy or mitophagy affect NLRP3 inflammasome activation, but NLRP3 inflammasome activation also dictates autophagy or mitophagy status. Appropriate inflammasome activation helps the organism cope with external metabolic stress. Excessive activation of NLRP3 inflammasome intensified the development of inflammatory products (95, 111). Autophagy is an essential process for the recycling and removal of damaged proteins and organelles. Autophagy-mediated degradation relies on lysosomes to remove double-membraned organelles. The acidic environment and proteases in the lysosome ensure normal autophagy. Autophagy can remove NLRP3 inflammasome activators (ROS and damaged mitochondria) and degrade NLRP3 inflammasome components, reducing inflammasome activation and the inflammatory response. Moreover, NLRP3 inflammasome signaling pathways can regulate the autophagic processes necessary to balance the required inflammatory response and prevent excessive and detrimental inflammation (112–114). Defective mitochondrial function is among the upstream signals that activate NLRP3 inflammasome. Myoung et al. found that global or brown adipocyte-specific deletion of *Pink1*, a Parkinson disease-related gene involved in mitophagy, induced BAT dysfunction, and obesity in mice (115). However, Zhang et al. suggested that *Nlrp3* may also serve as an upstream regulator for *Parkin*-mediated mitophagy in cardiomyocytes and is regulated by *iNOS* but unlikely mitochondrial ROS in *Akt2^−/−^* insulin resistance model (116). These data suggest that mitophagy and NLRP3 activation go both ways in regulating whole-body energy metabolism that might depend on certain cells or organs. Understanding the interrelation between these two essential biological processes is essential to comprehend the biological mechanisms and alleviate inflammation in obesity.

### 5.1 Autophagy Inhibits NLRP3 Inflammasome Activation

Recent studies have shown that autophagy mediates the activation of NLRP3 inflammasome. Activators of NLRP3 inflammasome can be removed by autophagy. Giordano et al. found that the increase in cholesterol crystals and accumulation of calcium and ROS in hypertrophic adipocytes in obesity triggered NLRP3 inflammasome with subsequent massive activation of Caspase-1 in sWAT and vWAT (117). Zhou et al. demonstrated that mitochondrial ROS was associated with NLRP3-dependent Caspase-1 activation and IL-1β release in monocytes and macrophages (118). 3-Methyladenine inhibited autophagy, which led to mitochondrial accumulation, generated ROS, and activated NLRP3 while enhancing inflammasomes. These inflammatory responses were reversed by ROS scavengers (118). Consistently, rapamycin (sirolimus) induced autophagy and suppressed the production of IL-1β and Caspase-1 activation (119). Disruption of autophagy could lead to the accumulation of damaged mitochondria. Autophagy maintained mitochondrial homeostasis by removing ROS produced by damaged mitochondria (118, 120). One study showed that deficiencies in mitochondrial clearance led to an increase in *Nlrp3* inflammasome and brown AT dysfunction, which could be reversed through *Nlpr3* deletion in *Pink1^-/-^* mice (115).

Inflammation in AT was improved by an interaction between adipocytes and macrophages with enhanced autophagy. Sirtuin 3 (SIRT3), an NAD+-dependent deacetylase, played an essential role in regulating macroautophagy and lipid metabolism. Sirtuin 3 regulated the 3T3-L1-mediated differentiation of adipocytes and activated the AMP-activated protein kinase-unc-51-like kinase 1 pathway in mature adipocytes by macroautophagy (121). The overexpression of *Sirt3* inhibited *Nlrp3* inflammasome by reversing mitochondrial dysfunction and activating AMPK in macrophages of vWAT. Moreover, activated autophagy attenuated inflammatory responses induced by the conditioned medium from macrophage in adipocytes and blocked the migration of macrophages toward adipocytes. This evidence suggested that autophagy regulated the activation of inflammasomes in a positive way.

### 5.2 Autophagy Targets NLRP3 Inflammasome Components

**Figure 2:**
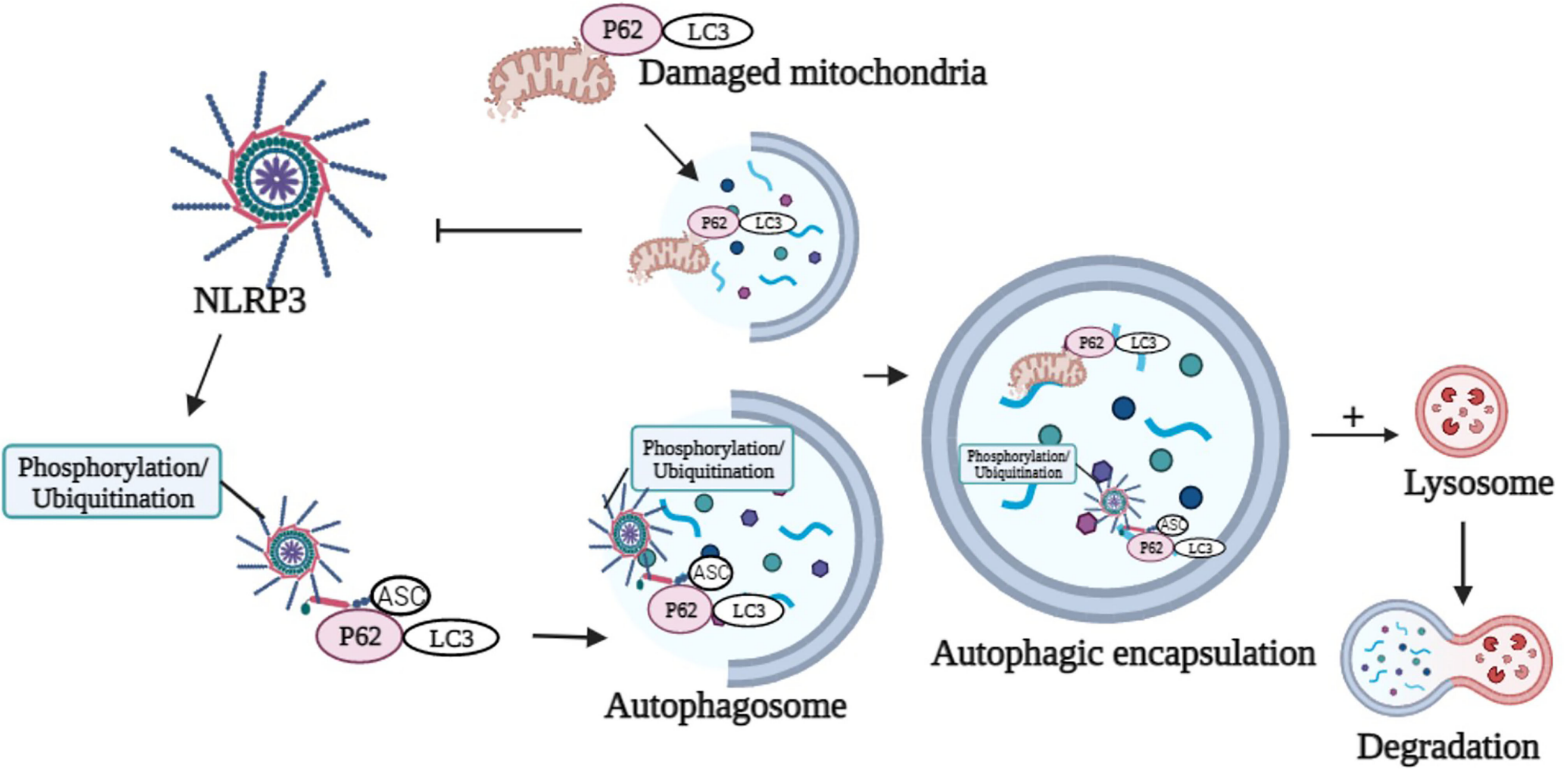
Graphical representation of p62 in the modulation of NLRP3 activation and degradation. p62 binds damaged mitochondria to reduce NLRP3 inflammasome activation. Phosphorylation or ubiquitination of NLRP3 interacts with p62 in an ASC-dependent manner and sequesters in phagosomes for degradation. Image created with BioRender.com.

P62 was a ubiquitinated degradation substrate associated with autophagy. LPS induced NF-κB-dependent p62 expression in macrophages. P62 was recruited to the damaged mitochondria upon NLRP3 activation. P62 bound damaged mitochondria that underwent *Parkin*-dependent clearance and reduced *Nlrp3* inflammasome activation in macrophages (122) (**Figure 2**). The possible mechanism was that inflammasome activation prevented p62-dependent degradation of inflammasome components. p62 recognized the adaptor protein ASC, an NLRP3 inflammasome component, which colocalized with autophagosomes. This result indicated that *Nlrp3* inflammasome could be engulfed and degraded by autophagosomes. Autophagy inhibition and targeted p62 inhibition markedly enhanced NLRP3 inflammasome activation (123). Recently, it was proposed that phosphorylation of NLRP3 is inactivated in an autophagy-dependent manner (124). Phosphorylated NLRP3 interacted with p62 in an ASC-dependent way and then was sequestered in phagosomes for degradation (124) (**Figure 2**). Modification of NLRP3 inflammasome (e.g., phosphorylation and ubiquitination) and subsequent autophagic encapsulation prevented excessive inflammatory responses (123, 124).

In addition, autophagy controlled the production of IL-1β by targeting pro-IL-1β for lysosomal degradation (**Figure 1**). Harris et al. reported that rapamycin enhanced autophagy, induced the degradation of pro-IL-1β and blocked the secretion of mature cytokines in macrophages. Inhibition of autophagy promoted the processing and secretion of IL-1β (114). Zhang et al. observed that autophagy could degrade IL-1β during the maturation of autophagosomes (125). Similarly, inhibiting autophagy in THP-1 cells reduced IL-1β secretion and increased intracellular IL-1β levels following LPS stimulation (126). The regulation of IL-1β release by autophagic mechanisms is complex and requires further investigation, depending on conditions such as cell type, inflammasome activators, autophagy inducers, or autophagy inhibitors.

### 5.3 NLRP3 Inflammasome Regulates Autophagy in AT

The status of autophagy or mitophagy may depend on inflammasomes. It was discovered that NLRP3 inflammasome enhanced autophagy initiation by interacting with Beclin-1 (127). NLRP3 acted as a regulator of autophagy. In THP-1 cells, overexpression of NLRP3 inflammasome enhanced autophagy and expression of the LC3-II protein (128). Similarly, silencing NLRP3 decreased autophagy and decreased the conversion of LC3-I to LC3-II (129). In addition to NLRP3, Caspase-1 has been shown to control the autophagic process *via* substrate cleavage (130, 131). Yu et al. found that activating NLRP3 inflammasome resulted in Caspase-1-dependent mitochondrial arrest in macrophages, resulting in the buildup of mitochondrial DNA and defective mitochondria. *Caspase-1*-mediated *Parkin* cleavage enhanced Caspase-1-dependent mitochondrial clearance from macrophages during inflammasome activation (132). Overall, inflammasome activation of mature adipocytes are rarely studied. It is worth investigating whether activating NLRP3 inflammasome affects autophagy initiation in mature adipocytes.

## 6 Conclusions and Remarks

The review summarized the current studies and potential mechanisms associated with autophagy and NLRP3 inflammasome in AT inflammation. Although hyperplasia and hypertrophy of adipocytes in AT are vital determinants in the progress of obesity, proinflammatory cytokine secretion and immune cell migration are the driving forces for systemic inflammation and insulin resistance, promoting alterations in (suppressed or enhanced) autophagy and NLRP3 inflammasome. Although clinical trials and animal models evaluated autophagy and inflammatory levels in isolated AT components, challenges still need to be overcome before targeted therapeutics will be clinically helpful for controlling obesity. First, it is relatively difficult to evaluate autophagic activity at a specific stage of growth and development in humans. At present, the ATG-knockout model and the evaluation of overall autophagic activity mainly come from animal research. Second, there are differences in insulin resistance, basic metabolic rates and antioxidant capacity among obese individuals in different metabolic states, resulting in inconsistent levels of basic inflammation. A unified evaluation of the relevant indicators of the level of inflammation is needed. Third, the body is in a state of dynamic equilibrium. Moderate autophagy and inflammation reflect AT adjustment to external energy, which is beneficial for reducing ectopic lipotoxicity. When studying autophagy modulators and inhibitors of NLRP3 inflammasome activation, the effects on the total metabolic capacity of the organism should be considered.

In conclusion, targeting autophagy and NLRP3 inflammasome as therapeutic strategies is beneficial in managing AT inflammation and obesity-related complications. Further studies should manipulate the exact pathways associated with altered autophagy and activated inflammasomes, leading to possible treatments for patients suffering from obesity.

## Author Contributions

LZ wrote the manuscript. LL designed the topic and critically revised the work. All authors contributed to the article and approved the submitted version.

## Funding

This study was funded by the National Natural Science Foundation of China [grant numbers 81270956 and 81470577].

## Conflict of Interest

The authors declare that the research was conducted in the absence of any commercial or financial relationships that could be construed as a potential conflict of interest.

## Publisher’s Note

All claims expressed in this article are solely those of the authors and do not necessarily represent those of their affiliated organizations, or those of the publisher, the editors and the reviewers. Any product that may be evaluated in this article, or claim that may be made by its manufacturer, is not guaranteed or endorsed by the publisher.
